# The great divide: drivers of polarization in the US public

**DOI:** 10.1140/epjds/s13688-020-00249-4

**Published:** 2020-10-28

**Authors:** Lucas Böttcher, Hans Gersbach

**Affiliations:** 1 Department of Computational Medicine, UCLA, Life Sciences Bldg., Box 951766, Los Angeles, US; 2 Institute for Theoretical Physics, ETH Zurich, Wolfgang-Pauli-Str. 27, 8093 Zurich, Switzerland; 3 Center of Economic Research, ETH Zurich, Zürichbergstrasse 18, 8092 Zurich, Switzerland

**Keywords:** Political polarization, Markov chains, Bayesian inference

## Abstract

Many democratic societies have become more politically polarized, with the U.S. being the main example. The origins of this phenomenon are still not well-understood and subject to debate. To provide insight into some of the mechanisms underlying political polarization, we develop a mathematical framework and employ Bayesian Markov chain Monte-Carlo (MCMC) and information-theoretic concepts to analyze empirical data on political polarization that has been collected by Pew Research Center from 1994 to 2017. Our framework can capture the evolution of polarization in the Democratic- and Republican-leaning segments of the U.S. public and allows us to identify its drivers. Our empirical and quantitative evidence suggests that political polarization in the U.S. is mainly driven by strong political/cultural initiatives in the Democratic party.

**Electronic Supplementary Material:**

The online version of this article (10.1140/epjds/s13688-020-00249-4) contains supplementary material.

## Introduction

Political polarization is on the rise in many democratic societies [1–6], and yet the causes of this relatively recent development are not well-understood. In the U.S., political polarization, in terms of ideological distance between Republicans and Democrats, has been growing significantly in recent years, so that it is now less likely to find a liberal Republican or a conservative Democrat than it was many years ago [2, 6]. Several explanations for this finding have been put forward, including the increasing influence of new media and the Internet, the rising income inequality, elite polarization, and demographic changes [4, 7, 8]. However, the growing use of the Internet, for instance, might not suffice to explain the observed polarization effects, because polarization is largest among demographic groups that are least likely to use the Internet and social media [3].

**Figure 1:**
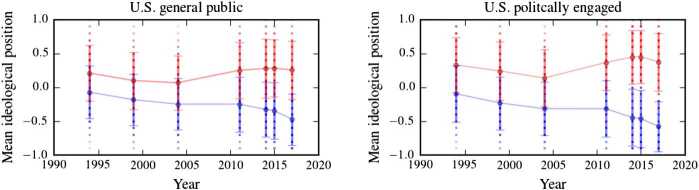
Polarization in the U.S. public. We show the mean-ideological position (i.e., the mean of the ideology distributions provided in [6]) of the Democratic- (blue) and Republican-leaning (red) segments of U.S. public from 1994 to 2017. Error bars indicate the observed standard deviation in each year. It is evident that polarization has been increasing in the past 15 years. The plotted data are based on a survey conducted by Pew Research Center (see [6] for details)

We study how the spreading of political and cultural ideas within populations that lean towards Democrats or Republicans can explain the evolution of political polarization, as observed in empirical data (see Fig. 1). In this context, mathematical models are able to offer insights into the dynamics of opinion formation, polarization, and related spreading processes [9–20]. To help quantify and characterize empirically-observed polarization trends (see Fig. 1), we develop a mathematical framework of political change based on (i) individuals’ diffusion from one ideological position to adjacent ones, and (ii) targeting of certain groups of individuals by *influential actors* who spread their ideas to coalesce around political/cultural positions (henceforth simply called “initiatives”). Theoretical motivation for the interaction of adjacent opinion groups in process (i) comes from related models of opinion formation that are based on the assumption that an exchange of views on a certain topic occurs if the corresponding opinions are not too different (i.e., only involve “nearest neighbors” in opinion space) [21]. We show in this paper that this parsimonious modeling approach leads to good agreement between simulation and empirical data. The term “influential actor” in process (ii) is typically used in the political economy literature and refers to political elites [22], opinion leaders of political interest groups and activists (see [23] for a theory of parties in which interest groups and activists are the key actors), and incumbent candidates of larger parties [24] that have an impact on, at least, some voter groups. More broadly, influential actors can be individuals or groups of individuals, with a particular political or cultural interest, whose ideas impact some voter segments [8, 25].

One current problem in social-influence modeling is that most models are *untested*. This is pointed out directly in the abstract of [21]: “More empirical work is needed testing and underpinning micro-level assumptions about social influence as well as macro-level predictions.” Our work addresses this issue by proposing a parsimonious DeGroot-like model [26] of opinion formation and demonstrating that such a model is able to capture the evolution of empirically-observed opinion polarization in the US public with very few parameters.

We demonstrate how to use Bayesian Markov chain Monte-Carlo (MCMC) and information-theoretic methods in conjunction with opinion-formation models and corresponding empirical data to assess and analyze levels and changes of ideology distributions in the United States. We find that a single parameter suffices to describe the evolution of polarization trends over the last 20–30 years and we identify this polarization measure [27] with the notion of *initiative impact*. This measure enables us to quantify relative changes in ideology distributions between Democratic- and Republican-leaning segments of U.S. society over time. Our results suggest that the recent polarization in the U.S. public, which took place during the Obama and Trump administrations (see Fig. 1), seems to be mainly driven by strong political/cultural initiatives in the Democratic party. Prominent examples of such initiatives are the Affordable Care Act, policy proposals involving higher tax rates on individuals with high income or wealth, tighter gun control, and same-sex marriages, as we will discuss in more detail below.

## The model

In this section, we first define a general and abstract Markov chain model to mathematically capture empirically observed polarization trends (see Fig. 1). Our model describes a (macroscopic) set of different opinion classes. Empirically, these opinion classes correspond to mappings of a high-dimensional feature space (e.g., self-identification with a certain political party, views on certain political issues) to a single-number metric. We briefly describe the update dynamics and then focus on the characterization of the stationary distribution.

### Definition of the ideology chain

We proceed in three steps to mathematically describe initiatives and the diffusion of individuals from one ideological position to adjacent ones, with step 1 developed in Sects. 2.1 and 2.2, step 2 in Sect. 2.3, and step 3 in Sect. 2.4.

In the first step, we consider a one-dimensional chain which consists of *N* different states denoted by *i* ($i\in \{1,\dots,N\}$). We use $X_{i}$ to denote the fraction of society in state *i*, and hence $\sum_{i=1}^{N} X_{i} = 1$. Next, we map the index *i* to an *ideological position* $x\in [-1,1]$ according to $x=2 (i-1)/(N-1)-1$. These positions represent the political spectrum in the following way: Very liberal individuals are located at the beginning of the chain ($i=1$, $x=-1$), whereas strongly conservative individuals are found at the opposite side ($i=N$, $x=1$). We next consider the evolution of a hypothetical society in discrete time. We interpret $X_{i}^{n}$ as the fraction of voters of type *i* at time step $n\in \mathbb{N}$. For every *n*, it holds that

1 $$ \sum_{i=1}^{N} X_{i}^{n}=1 $$

as a normalization condition. We employ a simple birth-death queue [28] of social interactions and assume that individuals may change their ideological position via interactions with their ideological neighbors. At the aggregate level, in a particular time step, we assume that transitions occur from state *i* to its nearest neighbors ($i\rightarrow i+1$ and $i\rightarrow i-1$) with some probabilities $p_{i}\in (0,1)$ and $q_{i-1}\in (0,1-p_{i})$. For the moment, these transition probabilities are taken as given and will be estimated later. At the boundaries of the opinion chain, the probability of becoming more ideologically extreme is zero ($q_{0}=p_{N}=0$). The probability of staying at a certain ideological position *i* is given by $r_{i}=1-p_{i}-q_{i-1}$. Together, these probabilities form the transition matrix *P*, with the following entries:

2 $$ P_{i i-1}=q_{i-1},\qquad P_{i i} = r_{i},\quad \text{and}\quad P_{i i+1}=p_{i}. $$

The probabilities in each row sum up to one, i.e. $\sum_{j=0}^{3} P_{i i-1+j}=1$.

**Figure 2:**
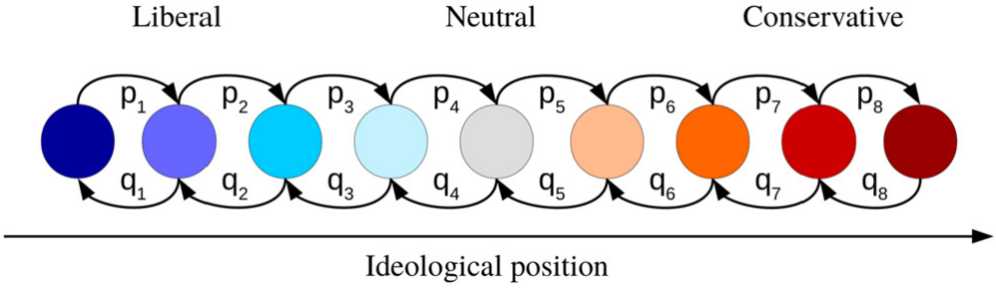
Polarization model. In our model, the political spectrum consists of *N* different states and is divided in three groups: liberal, neutral, and conservative. For illustrative purposes, we set $N=9$ in this example. The transition probabilities are denoted by $\{p_{i}\}_{i\in \{1,\dots,9\}}$ and $\{q_{i}\}_{i\in \{1,\dots,9\}}$

There exist different ways of motivating and microfounding our opinion-formation model. First, the mathematical structure of our model is similar to DeGroot’s [26] Markov-chain description of “social learning” in a group of communicating individuals. Second, the macroscopic distributions of opinions that we observe in our model can be recovered in random matching and bounded confidence models [29, 30] in which individuals adopt sufficiently close opinions through communication (see [31] for a comprehensive account on how such micro-level assumptions in a social network turn into macro-level implications and [32] for a general theory about such social interactions). After every meeting, individuals update their opinion and may switch to their partner’s opinion with some probability. Equivalently, individuals change their opinion if they meet a sufficient number of people with alternative opinions [17, 18, 33]. The probabilities $p_{i}$ and $q_{i}$ can then be interpreted as the resulting parameters at the aggregate level.^1^ We show an example of an ideology chain with $N=9$ states in Fig. 2. For the sake of clarity, we do not include self-loops described by $r_{i}$ in this figure.

Next we focus on the dynamics of the model to account for the diffusion of individuals from one ideology to adjacent ones. The initial values of all states are given by $X_{i}^{n=0}=X_{i}^{0}$. We use $X^{0}= (X_{1}^{0},\dots,X_{N}^{0} )$ to denote the row vector of all initial states. The time evolution of the ideology distribution is then described by $X^{0} P^{n} = X^{n}$.

### Stationary distribution

To determine the stationary ideology distribution, we formulate the update rule of state $X_{i}^{n}$ and find

3 $$ X_{i}^{n+1}=(1-p_{i}-q_{i-1})X_{i}^{n}+q_{i} X_{i+1}^{n}+p_{i-1} X_{i-1}^{n}. $$

We are not considering periodic boundaries, and thus find for $i=1$,

4 $$ X_{1}^{n+1}=(1-p_{1})X_{1}^{n}+q_{1} X_{2}^{n}. $$

The Markov chain converges to a stationary distribution, which we denote by $X_{i}$ with $i\in \{1,\dots,N\}$. Based on Eq. (4), we obtain $X_{2}= (p_{1}/q_{1} ) X_{1}$. Furthermore, using Eq. (3) we find by induction that [34]

5 $$ X_{i+1}= X_{1} \prod_{j=1}^{i} \frac{p_{j}}{q_{j}}. $$

To satisfy the normalization condition of Eq. (1), we set $X_{1}=1$ and divide each state $X_{i}$ by $\sum_{i=1}^{N} X_{i}$. The stationary distribution $X=(X_{1},\dots,X_{N})$ is unique since the transition matrix *P* is irreducible and aperiodic [35]. Irreducibility follows from the fact that any state in the Markov chain can be reaced from any other state, and aperiodicity is satisfied because of $P_{ii}^{n} > 0$ for all $n\in \mathbb{N}$ [35].

The data that we show in Fig. 1 suggests that the ideological overlap between Democrats and Republicans was larger in the 1990s and early 2000s compared to the last 15 years. To capture the ideology distributions of Democratic- and Republican-leaning segments of the U.S. public with our model, we consider two opinion chains *A* and *B* in the subsequent sections, and then account for the impact of influential actors and their initiatives.

### Two populations

In the second step, we introduce two populations in which members influence each other regarding their ideological position. This allows us to examine how the distribution of ideologies among Democrats and Republicans evolves over time. Specifically, we consider two populations, *A* and *B*, with the corresponding stationary ideology distributions given by Eq. (5):

6 $$\begin{aligned} X^{A}_{i+1}=X^{A}_{1} \prod _{j=1}^{i} \frac{p^{A}_{j}}{q^{A}_{j}} \end{aligned}$$

and

7 $$\begin{aligned} X^{B}_{i+1}=X^{B}_{1} \prod _{j=1}^{i} \frac{p^{B}_{j}}{q^{B}_{j}}. \end{aligned}$$

### Influential actors

In the third step, we account for influential actors of both parties that inject political/cultural concepts—simply called *initiatives* in our paper—into one of the populations. The literature has identified the importance of such influential actors and their initiatives (see e.g. [8, 25]). Typically, initiatives increase the attractiveness of coalescing around a particular ideological position. They can also increase the cohesion within each population and the identity value of belonging to a population.

Mathematically, we describe the impact of influential actors on the two populations in terms of rescaling the transition probabilities of Eq. (2) by $\lambda _{A}$ and $\lambda _{B}$ at a particular point in time according to

8 $$ p_{i}^{A}\rightarrow p_{i}^{A}/\sqrt{ \lambda _{A}} \quad\text{and}\quad q_{i}^{A} \rightarrow q_{i}^{A} \sqrt{\lambda _{A}}. $$

For opinion group *B* (e.g., Republicans), the rates are modified as follows:

9 $$ p_{i}^{B}\rightarrow p_{i}^{B} \sqrt{ \lambda _{B}} \quad\text{and}\quad q_{i}^{B} \rightarrow q_{i}^{B} /\sqrt{\lambda _{B}}. $$

The interpretation of the scaling factor is as follows: A value of $\lambda _{A}>1$ and $\lambda _{B}>1$ means that an initiative in the populations A and B is introduced, which attracts individuals towards the left and right ends of the underlying ideology chains, respectively. The larger $\lambda _{A}$ and $\lambda _{B}$, the greater is the attractiveness of these initiatives. Based on Eqs. (8) and (9), we obtain the following modified stationary states:

10 $$ X^{A}_{i+1}=\lambda _{A}^{-i} X^{A}_{1} \prod_{j=1}^{i} \frac{p^{A}_{j}}{q^{A}_{j}} $$

and

11 $$ X^{B}_{i+1}=\lambda _{B}^{i} X^{B}_{1} \prod_{j=1}^{i} \frac{p^{B}_{j}}{q^{B}_{j}}. $$

The outlined multiplicative rescaling of the transition probabilities leads to a directly-interpretable modification of the stationary opinion distribution. If $\lambda _{B} > 1$, we obtain a stationary opinion distribution whose mean is shifted to the right compared to the case where $\lambda _{B} = 1$. Similarly, if $\lambda _{A} > 1$, the stationary opinion distribution moves towards the left. We thus refer to *λ* as the *initiative impact*.

When we move to the empirical application, we have to recognize that a particular value of $\lambda _{B}$ or $\lambda _{A}$, which we infer from the data, is open to different interpretations. On the one hand, it could represent changes in the way individuals communicate and influence each other, resulting in a shift regarding political/cultural views. On the other hand, it could represent the impact of new ideas (political or cultural) that affect the attractiveness of different ideological positions as we have outlined in our model.

While it is difficult to disentangle these two interpretations, we will interpret our findings in light of the second interpretation. We do this for two reasons: First, changes in communication, e.g. due to increased Internet use [3] cannot explain the rise in polarization, and [36] recently summarized that the evidence about whether social media increase political polarization is not conclusive. Second, there exists a series of legal acts and policy proposals that have been at the center of communication in the community leaning towards the Democratic party. Such legal acts include the Dodd–Frank Financial Reform Act and the Affordable Care Act. Examples of policy proposals are universal-health care programs, higher taxes on wealth, tighter gun control, and initiatives to slow down climate change. Opposition against tighter gun control, higher taxes, and same-sex marriages are examples of major ideas in the communities leaning towards the Republican party. These examples suggest that major new initiatives have been mainly introduced in the communities leaning towards the Democratic party. We examine whether the data are consistent with this interpretation. We also note that some of the initiatives such as same-sex marriage and abortion rights are culture-dependent since they concern norms and beliefs about how people should be able to live in families, groups and communities.

In the following sections, we show that the outlined rescaling approach is able to model empirically-observed opinion polarization in the U.S. public. In principle, we could also consider values of $\lambda _{A}$ and $\lambda _{B}$ that depend on the position in ideology space. It is, however, possible to capture a substantial part of the polarization effects with a constant value of $\lambda _{A}$ and $\lambda _{B}$, as shown in Sect. 3.2.

## Results

We now focus on the applications and implications of the polarization model introduced in Sect. 2. In Sect. 3.1, we discuss the onset of political polarization when influential actors in each party introduce new political ideas. We outline in Sect. 3.2 how our mathematical framework is able to capture a relevant amount of the polarization effects which have been observed in the U.S.-American public in the past 25 years. Once initialized, only the two initiative impacts $\lambda _{A}$ and $\lambda _{B}$ are necessary to describe these polarization effects. We use a Bayesian MCMC approach to learn the parameter distributions of $\lambda _{A}$ and $\lambda _{B}$ from empirical observations. Our results are consistent with a stronger polarization of the Democratic wing in the society compared to the Republican wing.

### Emergence of political polarization

**Figure 3:**
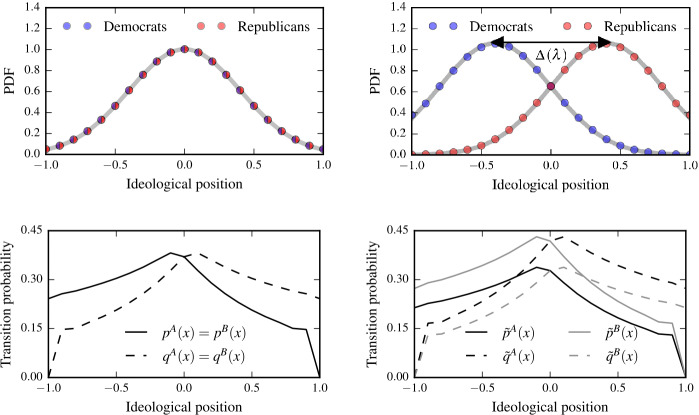
The emergence of political polarization. The top panels show an unpolarized (left panel) and a polarized (right panel) ideology distribution (synthetic/simulation data only). Democrats are represented by blue disks and Republicans by red disks. We use $\Delta (\lambda )$ to denote the absolute value of the difference between the shifts in the mean ideologies. The bottom panels show the corresponding transition probabilities which define the ideology distribution according to Eqs. (6) and (7) with $N=21$ states. We rescaled the probabilities in the right panel according to Eqs. (10) and (11) by setting $\lambda =\lambda _{A}=\lambda _{B}=1.13$

To study the emergence of political polarization in terms of our model as described in Sect. 2, we first consider an unpolarized society and then analyze the impact of influential actors. Let the ideology space be denoted by the interval $[-1,1]$. As initial ideology distributions, we consider normally-distributed (unpolarized) ideologies with mean $\mu =0$ and variance $\sigma ^{2}=0.16$, as illustrated in the upper left panel of Fig. 3. We note that a distribution of ideologies within a party does not uniquely determine the transition probabilities for the diffusion of ideas (see SI). The reason is that, according to Eqs. (6) and (7), only their fractions are relevant for the stationary distribution. To anchor meaningful transition probabilities, we take into account that voters with polar ideological positions are less likely to undergo a transition to more moderate ideological positions. In addition, we would expect larger transition probabilities in the more neutral ideology regime. These two properties anchor the transition probabilities. We show an example of the corresponding transition probabilities $p^{A}(x)$, $q^{A}(x)$, $p^{B}(x)$, and $q^{B}(x)$ in the lower left panel of Fig. 3. Also in the case of our empirical application in Sect. 3.2, the indeterminacy regarding the transition probabilities is resolved by taking the described effects into account.

We now incorporate the impact of influential actors on voter ideologies as described by Eqs. (10) and (11), and rescale the transition probabilities accordingly to obtain

12 $$\begin{aligned} \tilde{p}^{A}(x) = p^{A}(x)/\sqrt{\lambda },\qquad \tilde{q}^{A}(x) = q^{A}(x) \sqrt{\lambda }, \end{aligned}$$

and

13 $$\begin{aligned} \tilde{p}^{B}(x) = p^{B}(x) \sqrt{\lambda },\qquad \tilde{q}^{B}(x) = q^{B}(x)/ \sqrt{\lambda }, \end{aligned}$$

where we assumed $\lambda =\lambda _{A}=\lambda _{B}$. In the upper right panel of Fig. 3, we show that the outlined rescaling of the transition probabilities leads to shifted normal distribution with mean $\mu (\lambda )$ and an invariant variance. In this example, we set $\lambda =1.13$. This observation suggests that a rescaling of transition probabilities according to Eqs. (12) and (13) leads to a polarized ideology distribution. The corresponding transition probabilities and their rescaled versions are shown in the lower right panel of Fig. 3. As shown in the upper right panel of Fig. 3, it is possible to quantify polarization as $\Delta (\lambda )$, the absolute value of the difference between the shifts in the mean ideologies, which is $2 \mu (\lambda )$ in this example. Larger values of *λ* lead to an increase in $\Delta (\lambda )$. In other words, polarization is monotonically increasing with initiative impact.

### Polarization in the American public

After having outlined the basic polarization mechanism in our model, we now focus on the evolution of polarization as observed in empirical data on ideology distributions of US citizens from 1994 to 2017. The dataset that we use in this study is based on a Pew Research Center survey on political polarization [6]. Since 1994, Pew Research Center periodically conducts this survey by asking participants a set of 10 questions (e.g., “Should homosexuality be accepted by society?” or “Is good diplomacy the best way to ensure peace?”), which are traditionally associated with liberal/conservative views [6]. Conservative answers are recorded with a “+1” and liberal answers with a “−1”. The most conservative survey participants can reach a score of +10, whereas the most liberal value is −10. Note that we normalized the ideology scale to the interval $[-1,1]$ in our study.

Survey participants were also asked if they identify themselves as Democrats, Republicans, or neither and if they are politically engaged [37]. According to [37], persons with high political engagement are “registered to vote, always or nearly always vote, and in the past year have volunteered for or contributed to a campaign”.

All surveys were conducted by Pew Research Center either online with the Pew Research American Trends Panel or by Pew Research employees using a random digit sample of landline and cellphone numbers in the United States. People were contacted and surveyed by an interviewer in person or on the telephone (either landline or cellphone), and via the Internet and paper questionnaires (delivered in person or per mail). The usual sample size of Pew Research Center polls is about 1500 people, but may vary from survey to survey [38]. For a detailed overview about the survey methodology, see [39].

Ideology distributions are available for the Democratic- and Republican-leaning segments of the U.S. general and politically-engaged public. We initialize our opinion chain by using the empirical ideology distributions of 1994 to determine the transition probabilities as defined by Eq. (2) with a maximum-likelihood estimation. As in Sect. 3.1, we consider the case where the transition probabilities are monotonically increasing towards the center. After the initialization procedure, the parameters $\lambda _{A}$ and $\lambda _{B}$ are the only two free parameters in our model. They will describe the observed ideology distribution according to Eqs. (10) and (11). In the next step, we use a Bayesian MCMC approach to learn the distributions of the two parameters that best describe our data [40, 41]. The theoretical background is presented in the SI.

**Figure 4:**
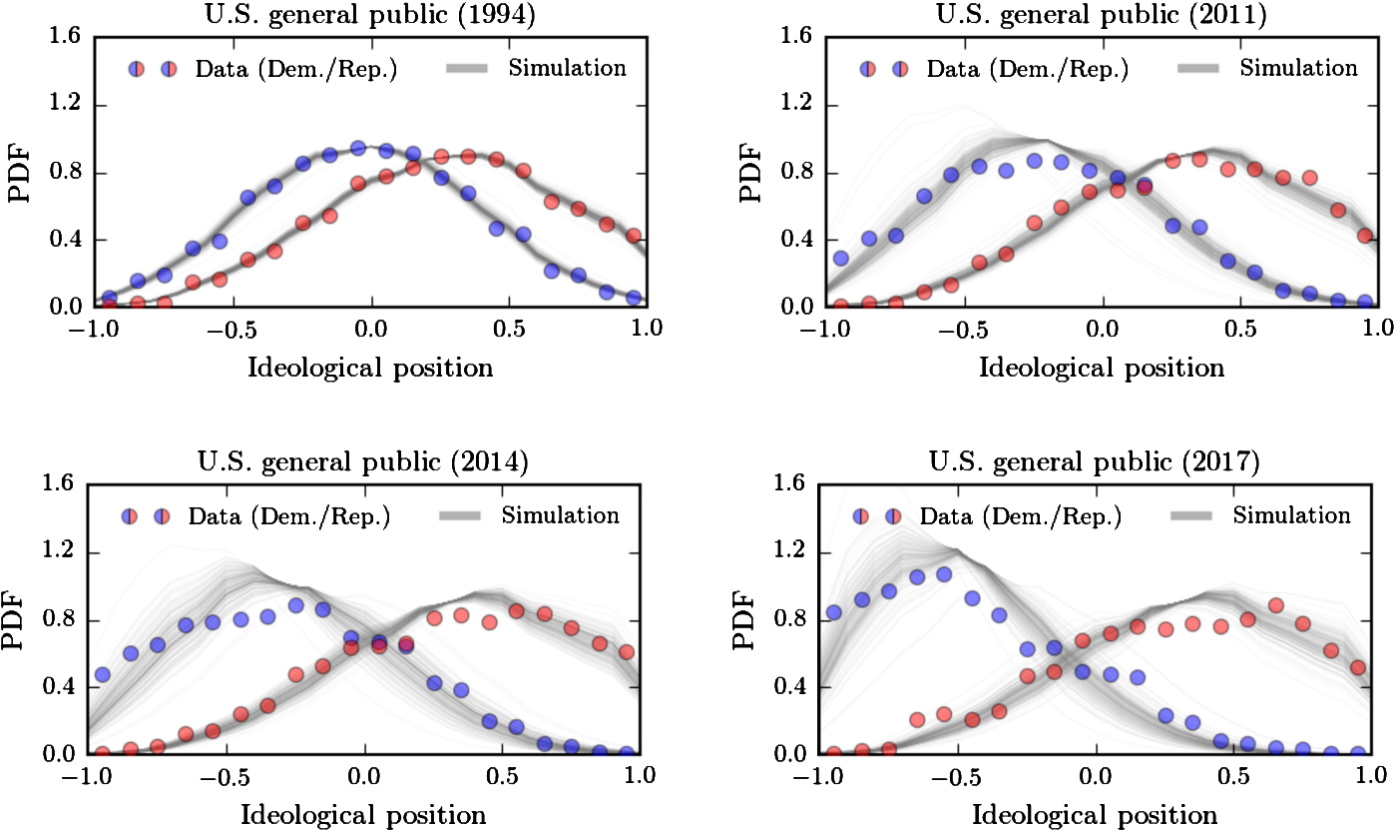
Polarization in the U.S. general public in comparison with our model. For the U.S. general public, we show the ideology distributions for Democrats (blue disks) and Republicans (red disks). The dataset is based on a survey conducted by Pew Research Center (see [6] for details). We initialized our model (grey solid lines) with the data of 1994 and only modified the distributions according to a transition probability rescaling as described by Eqs. (10) and (11)

**Figure 5:**
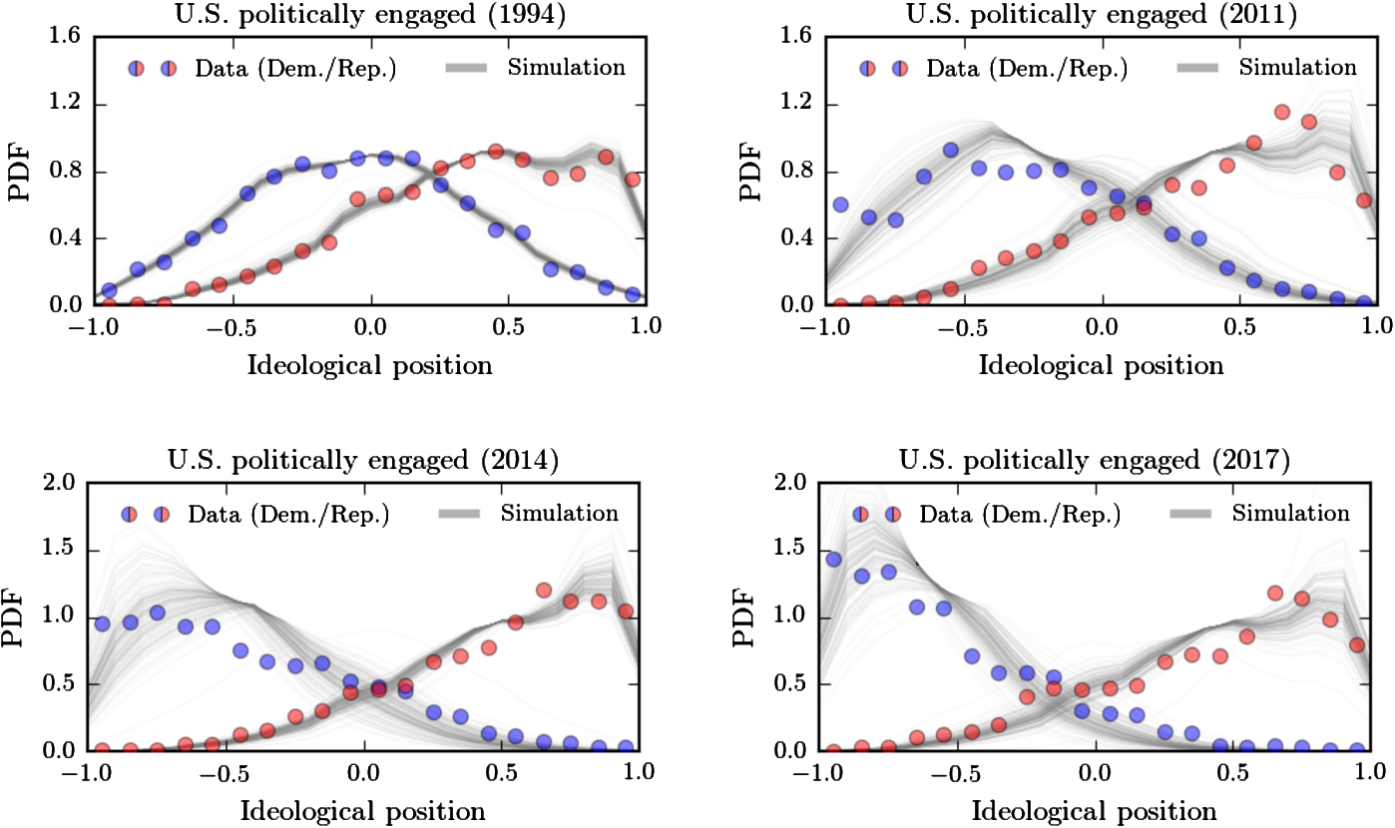
Polarization in the U.S. politically-engaged public in comparison with our model. For the U.S. politically engaged public, we show the ideology distributions for Democrats (blue disks) and Republicans (red disks). The dataset is based on a survey conducted by Pew Research Center (see [6] for details). We initialized our model (grey solid lines) with the data of 1994 and only modified the distributions according to a transition probability rescaling as described by Eqs. (10) and (11)

We show the empirical ideology distributions and corresponding simulations in Figs. 4 and 5. In 1994, the ideology distributions of Democrats and Republicans are almost Gaussian and centered around the origin. In subsequent years, polarization becomes more and more apparent. The division is much more drastic for politically-engaged citizens, compared to the general public. The plots also reveal that, after the initial transition probability estimation, our two-parameter model is able to capture the time evolution of the two ideology distributions quite well. Hence, a substantial portion of the complex ideology and identity formation process can be captured by a multiplicative rescaling of the transition probabilities according to Eq. (12).

**Figure 6:**
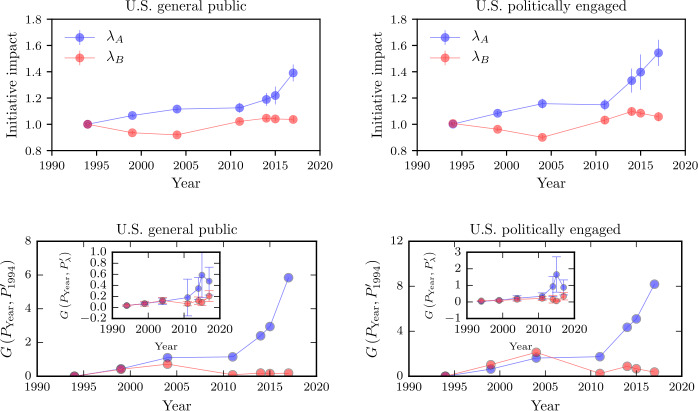
Initiative impacts and relative entropy over different years. In the upper panels, we show estimates of the initiative impact $\lambda _{A}$ and $\lambda _{B}$ as defined in Eqs. (10) and (11). We see that the polarization effects for self-identified Democrats are larger than for self-identified Republicans. We also compute the relative entropy usning Eq. (14) and show the results in the lower panels. $P_{\text{Year}}$, $P^{\prime }_{1994}$ and $P^{\prime }_{\lambda }$ are the distributions of the data in different years, of the model in 1994 and of our model in different years, respectively. Blue disks represent Democrats and red disks represent Republicans

For every year, we show the corresponding distributions of $\lambda _{A}$ and $\lambda _{B}$ in the SI in Fig. S2 and illustrate the time evolution of $\lambda _{A}$ and $\lambda _{B}$ in Fig. 6.

The initiative impact *λ* may be also interpreted as a polarization measure. Such an interpretation allows us to analyze the polarization dynamics in the U.S. more systematically. The data presented in the upper panels of Figs. 6 and S2 make clear that the Democratic initiative impact $\lambda _{A}$ increases substantially over time. The distributions of $\lambda _{A}$ are also getting broader, an effect which results from additional ideology-transition effects that we cannot describe by simply rescaling the initially-determined transition probabilities. Interestingly, the polarization behavior associated with the Republican initiative impact $\lambda _{B}$ differs significantly from the polarization dynamics in the Democratic-leaning segment of society. In fact, in 1999 and 2004, the values of $\lambda _{B}<1$ imply that Republicans are ideologically moving towards the center. Just after 2011, the values of $\lambda _{B}>1$ suggest that more right-wing initiatives have been transmitted. As a result, Republicans are moving to the right in the political spectrum. Our results clearly suggest, however, that polarization is mainly driven by initiatives acting on the Democratic wing of society.

In the last step, we compare differences between ideology distributions in each year using the relative entropy (or Kullback–Leibler divergence)

14 $$ G \bigl(P_{\text{Year}},P^{\prime } \bigr)=\sum _{x} P_{\text{Year}}(x) \ln \biggl[\frac{P_{\text{Year}}(x)}{P^{\prime }(x)} \biggr], $$

where $P_{\text{Year}}$ is the distribution of the empirical data in the respective year and $P^{\prime }$ is the ideology distribution of the model or a certain reference year. To directly quantify the ideology-distribution evolutions from 1994–2017, we determine the relative entropy of $P_{\text{Year}}$ with respect to the empirical data distribution $P^{\prime }_{1994}$ of 1994. The results are shown in the lower panels of Fig. 6. We again observe that the Democratic distributions deviate much more from those of 1994 compared to the Republican distributions—another indicator of a larger polarization effect in the Democratic wing of the society. The insets in the lower panels of Fig. 6 show the relative entropy of $P_{\text{Year}}$ with respect to the distribution of our model $P^{\prime }_{\lambda }$. The small values of $G (P_{\text{Year}},P^{\prime }_{\lambda } )$ indicate that our model captures the empirical distributions well.

## Conclusion

We developed a mathematical framework and used Bayesian MCMC and information-theoretic methods to analyze empirical ideology distributions of the Democratic- and Republican-leaning segments of the U.S.-American public. Our framework is based on two processes: varying strength of initiatives in each party, and the corresponding diffusion of these concepts in society.

The evolution of the observed ideology distributions is quite well captured by only two parameters, namely the two initiative impacts. Due to their ability to describe the increasing gap between ideology distributions in the U.S. public, we propose to use these parameters as polarization measures and possible indicator of social conflict [42], in line with other polarization/diversity metrics [27] such as those of Esteban and Ray [43], D’Ambrosio and Wolf [44], and Wang and Tsui [45].

Our empirical and quantitative evidence suggests that strong initiatives from the Democratic party were the main drivers of the great divide that emerged in recent decades between the Democratic- and Republican-leaning population. Future studies may use the proposed framework to quantify polarization trends in other countries.

## Electronic Supplementary Material

Below is the link to the electronic supplementary material.

Supporting information. (PDF 744 kB)

## Footnotes

1 An alternative foundation of the model is competition of *N* echo chambers. Individuals communicate in one echo chamber, but opinion leaders of echo chambers spread their views to adjacent echo chambers to increase their number of followers.
