# Supplementary material for: The great divide: drivers of polarization in the US public
Source: EPJ Data Sci. 2020 Oct 28;9(1):32. doi: 10.1140/epjds/s13688-020-00249-4 (PMC7591448; doi:10.1140/epjds/s13688-020-00249-4)
Supplement: Supplementary file 1 — Supporting information. (PDF 744 kB) [file 13688_2020_249_MOESM1_ESM.pdf]

SUPPORTING INFORMATION (SI)

The Great Divide: Drivers of Polarization in the US Public

Lucas Böttcher[1,2,3,*] and Hans Gersbach[3]

[1] *Computational Medicine, University of California, Los Angeles, 90095-1766, Los Angeles, United States*

[2] *Institute for Theoretical Physics, ETH Zurich, 8093, Zurich, Switzerland*

[3] *Center of Economic Research, ETH Zurich and CEPR, 8092 Zurich, Switzerland*

(Dated: October 23, 2020)

* lucasb@ucla.edu

I. DETERMINING TRANSITION PROBABILITIES

According to Eqs. (10) and (11) in the main text, the transition probabilities p_i^A, q_i^A, p_i^B, and q_i^B at the ideological positions $i \in \{1, \dots, N\}$ are not uniquely determined by a given ideology distribution. The reason is that in Eqs. (10) and (11) only the fractions p_i/q_i are relevant for determining the stationary distribution of the ideology chain. To identify meaningful transition probabilities, we account for the fact that voters with polar ideological positions are less likely to adopt more moderate ideological positions. Furthermore, we expect larger transition probabilities in the neutral ideology regime. As initial values, we use the transition probabilities

$$p_i = \begin{cases} 0.2 \cdot \alpha^i & \text{for } i < N/2, \\ 0.2 & \text{otherwise,} \end{cases} \tag{S1}$$

and

$$q_i = \begin{cases} 0.2 \cdot \alpha^i & \text{for } i > N/2, \\ 0.2 & \text{otherwise.} \end{cases} \tag{S2}$$

We consider $N = 20$ states and we set $\alpha = 1.1$ as starting values. The corresponding probability curves for the chosen starting values are shown in the upper left panel of Fig. S1. We discuss the influence of noise on transition probabilities and the resulting opinion distributions in Sec. IV. After this initialization, we use a least-square optimization to find the transition probabilities that describe the Gaussian ideology distribution shown in Fig. 3 of the main text. The corresponding transition probabilities and fractions p_i/q_i are shown in the middle left and bottom left panel of Fig. S1, respectively. In the right panels of Fig. S1, we show that starting from another initial distribution may lead to different transition probabilities, but to the same fractions p_i/q_i. We again note that according to Eqs. (10) and (11) in the main text, a given ideology distribution is uniquely determined by the fractions p_i/q_i.

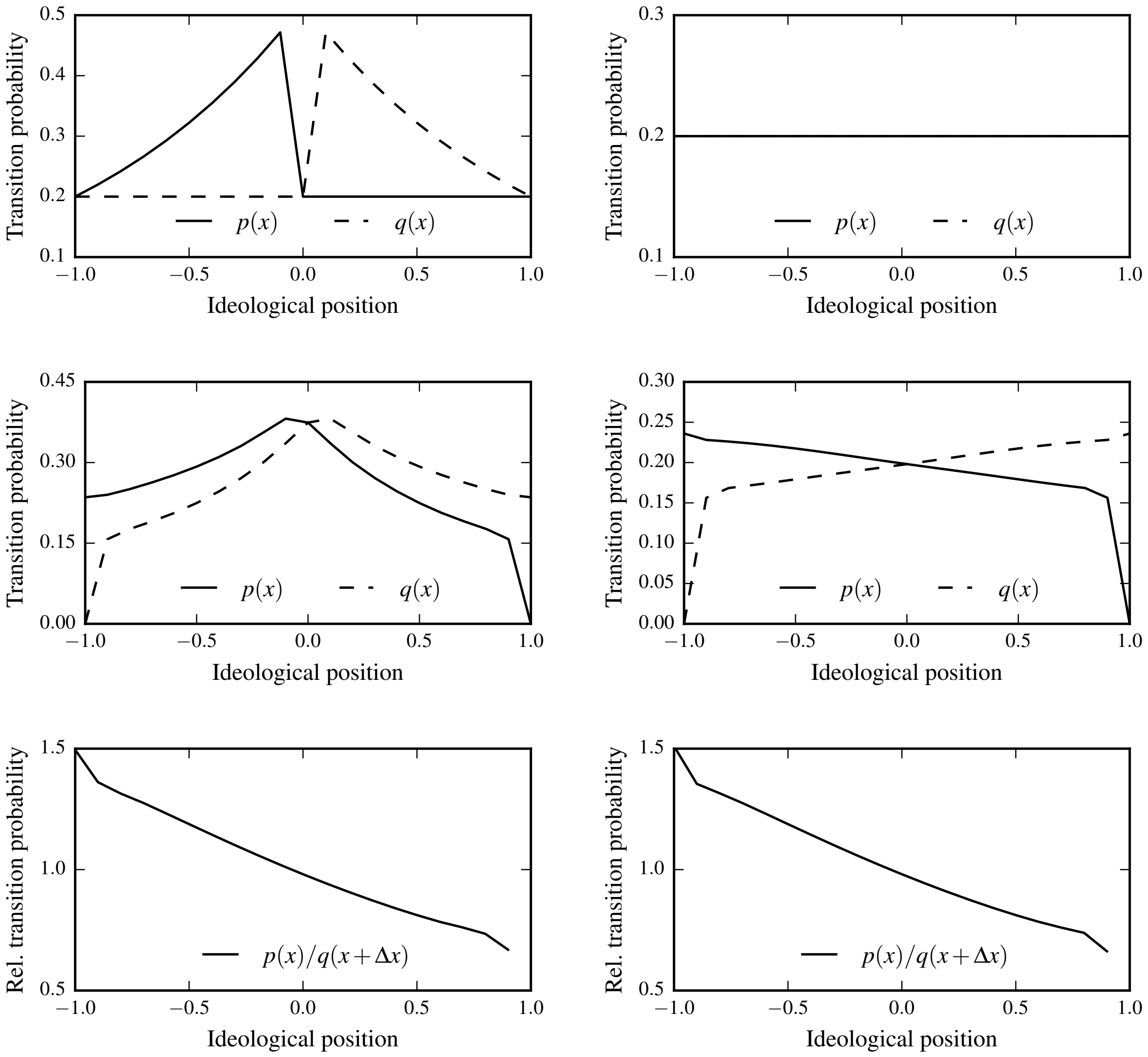

Figure S1. **Determining transition probabilities.** We numerically determine the transition probabilities describing the Gaussian ideology distribution shown in Fig. 3 in the main text. In the top left panel, we start from initial transition probabilities as defined by Eqs. (S1) and (S2) and use a least-square optimization method to determine the transition probabilities of the Gaussian ideology distribution. The results are shown in the middle left panel and the corresponding fraction $p(x)/q(x+\Delta x)$ is shown in the bottom left panel. In the right panels, we repeat the same procedure for an initially uniform probability distribution. The fractions $p(x)/q(x + \Delta x)$ are unaffected by this choice. Note that Δx corresponds to $2/(N - 1)$ according to the definitions in the main text. In all shown plots, we use the convention that q_{i+1} is the probability of an opinion change from state $i + 1$ to state i.

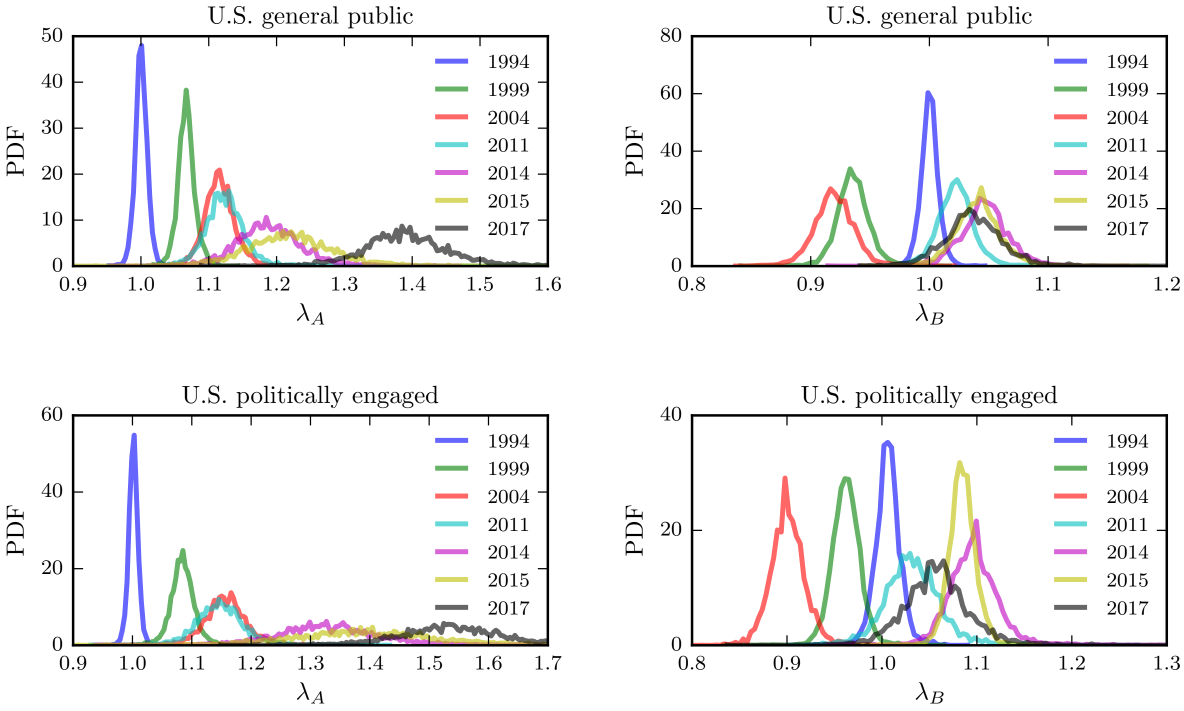

Figure S2. **Initiative strength distributions across time.** We determine the probability density functions (PDFs) of the initiative impacts λ_A and λ_B using Bayesian Markov Chain Monte-Carlo. The values of λ_A become larger over time, whereas the values of λ_B are more concentrated around unity.

II. INITIATIVE STRENGTH DISTRIBUTIONS

In the main text we have outlined that the initiative impact λ_A of self-identified Democrats grew in the period from 1994 to 2017. In contrast, the initiative impact λ_B of self-identified Republicans remained much more concentrated around unity and grew only little in the last years. In Fig. 6 of the main text, we show the mean values and standard deviations of λ_A and λ_B. These quantities were obtained from the corresponding Bayesian Markov Chain Monte-Carlo distributions of Fig. S2. These distributions also show that the initiative impact λ_A increases with time. In addition, we observe that the width of the distribution of λ_A is getting larger over time, because not all features of the ideology-distribution evolution can be captured using a single parameter, namely the initiative impact. This observation is also reflected by the increasing standard deviation in Fig. 6 in the main text. For self-identified Republicans, the corresponding distributions of λ_B are more concentrated around the initial

distribution of 1994. The broadening of the associated distributions' widths is also less pronounced.

III. BAYESIAN MARKOV CHAIN MONTE CARLO

To compute the distribution of the parameter set $\theta \in \{\lambda_A, \lambda_B\}$ of the ideology chain as defined by Eqs. (10) and (11) in the main text, we have to determine the probability distribution $P(\theta|D)$ given our data D on the distribution of ideologies of self-identified Democrats and Republicans. According to Bayes' theorem, we express the posterior distribution

$$P(\theta|D) \propto P(D|\theta)\, P(\theta)\,, \tag{S3}$$

in terms of the likelihood function $P(D|\theta)$ and the prior parameter distribution $P(\theta)$. We use a uniform prior distribution (between 0.5 and 2.0) and a Gaussian likelihood function

$$P(D|\theta) \propto \exp\left(-\frac{E^2}{2\sigma^2}\right), \tag{S4}$$

of the error E with zero mean and variance σ^2. Next, we use the prediction of our model $X_i(\theta)$ for a given parameter set θ and the actual ideology distribution data D_i ($i \in \{1, 2, \ldots, 20\}$) to compute the least square error

$$E^2 = \sum_{i=1}^{20} \left[D_i - X_i(\theta)\right]^2 \tag{S5}$$

as our error estimate in Eq. (S4) [1]. For a given prior parameter distribution $P(\theta)$, we compute the posterior distribution $P(D|\theta)$ using Bayesian Markov chain Monte-Carlo sampling with a Metropolis update scheme [1, 2]. After initializing the parameter vector with θ^0 drawn from the prior distribution $P(\theta)$, the nth iteration of the algorithm is defined by the following updates:

1. A new parameter set θ^* is drawn from the proposal distribution $J(\theta^*|\theta^n)$ (see below).

2. The acceptance probability for θ^* is computed according to (Metropolis algorithm)

$$r = \min\left(\frac{P(\theta^*|D)}{P(\theta^n|D)}, 1\right) = \min\left(\frac{P(D|\theta^*)\, P(\theta^*)}{P(D|\theta^n)\, P(\theta^n)}, 1\right). \tag{S6}$$

3. Draw a random number $\epsilon \sim U(0, 1)$ and set

$$\theta^{n+1} = \begin{cases} \theta^*, & \text{if } \epsilon < r, \\ \theta^n, & \text{otherwise.} \end{cases} \tag{S7}$$

For a uniform prior distribution, this update procedure implies that a new parameter set is always accepted if the new likelihood function value is greater than or equal to that of the previous iteration (i.e., for $P(D|\theta^*) \geq P(D|\theta^n)$). For the described Metropolis algorithm, the proposal distribution $J(\theta^*|\theta^n)$ must be symmetric. According to Ref. [2], a multivariate Gaussian distribution

$$J(\theta^*|\theta^n) \sim N\left(\theta^n|\tilde{\lambda}^2\Sigma\right) \tag{S8}$$

may be used as proposal distribution. The covariance matrix is denoted by Σ and the corresponding scaling factor by $\tilde{\lambda}$. Every 500 iterations, the covariance matrix and the scaling factor are updated, using the following update procedure [1, 2]:

$$\begin{aligned} \Sigma_{k+1} &= p\Sigma_k + (1-p)\Sigma^*, \\ \tilde{\lambda}_{k+1} &= \tilde{\lambda}_k \exp\left(\frac{\alpha^* - \hat{\alpha}}{k}\right), \end{aligned} \tag{S9}$$

where Σ^* and α^* are the covariance matrix and the acceptance rate of the last 500 iterations, respectively. The remaining parameters are $p = 0.25$, $\Sigma_0 = \mathbb{I}$, and $\lambda_0 = 2.4/\sqrt{d}$, where $\mathbb{I}$ is the $d \times d$ identity matrix and d the number of estimated parameters. The target acceptance rate is [2]

$$\hat{\alpha} = \begin{cases} 0.44 & \text{if } d = 1, \\ 0.23 & \text{otherwise.} \end{cases} \tag{S10}$$

To evaluate Eq. (S6), we have to compute the least square error for the new proposed parameter set θ^* according to Eq. (S5) to obtain the likelihood function value $P(D|\theta^*)$ based on Eq. (S4). For implementing a convergence test, we set the variance of our likelihood distribution to $\sigma^2 = 1/10$. Convergence is measured in terms of the Gelman-Rubin Test [2]. Therefore, we consider four independent Markov Chains. Every chain is initialized with a different random parameter set that is drawn from the corresponding prior distributions. The posterior parameter distribution is considered to be converged if the variance between the chains is similar to the variance within the chain (Gelman-Rubin Test). In Fig. 3, we illustrate the convergence behavior. We first let the four chains evolve for 10^4 iterations to then apply the Gelman-Rubin Test. After reaching convergence, we generate 10^4 more samples without updating the covariance matrix. These samples define the posterior parameter distribution.

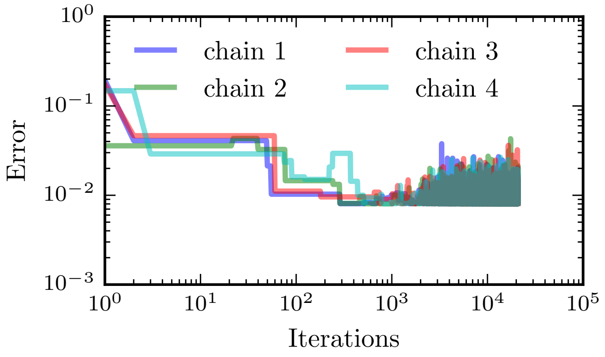

Figure S3. **Convergence of Bayesian Markov Chain Monte Carlo parameter estimation.** We show the evolution of the error as defined by Eq. (S5) for four different chains with different initial conditions. The algorithm is considered to be converged to the posterior distribution if the variance *between* the chains is similar to the variance *within* the chains (which is precisely captured by the Gelman-Rubin Test) [2]. We first let the system evolve for 10^4 iterations to then apply the Gelman-Rubin Test. After passing this convergence test, we generate 10^4 more samples without updating the covariance matrix to obtain the final parameter distributions. Fluctuations that appear for more than 10^3 iterations are a result of sampling values of λ_A and λ_B from the true distributions (cf. Fig. S2).

IV. INFLUENCE OF NOISE

To examine the robustness of the transition-probability rescaling, we allow that the transition probabilities p_i^A, q_i^A, p_i^B, and q_i^B of the discrete locations $i \in \{1, \ldots, N\}$ may be subject to noise. To analyze the propagation of noise effects in the transition-probability rescaling process, we consider relative fluctuations in the initial transition probabilities by an amount of $\epsilon \in [0,1]$. For example, we map each value of p_i^A to a value in the interval $\left[(1-\epsilon)p_i^A, (1+\epsilon)p_i^A\right]$. For a uniformly distributed random variable $u \sim \mathcal{U}(0,1)$, we map p_i^A to $[1+\epsilon(2u-1)]\, p_i^A$ and apply the same mapping to the other transition probabilities. In Fig. S4, we show two examples of noise influences ($\epsilon = 0.05$ and $\epsilon = 0.1$) on the initial transition probabilities (left panels) and the rescaled ones (right panels). In both cases, the noisy data (blue and red disks) are still in good qualitative agreement with the unperturbed Gaussians (grey solid lines). This suggests that the transition-probability rescaling is also applicable for noisy data if the noise influence is not too large.

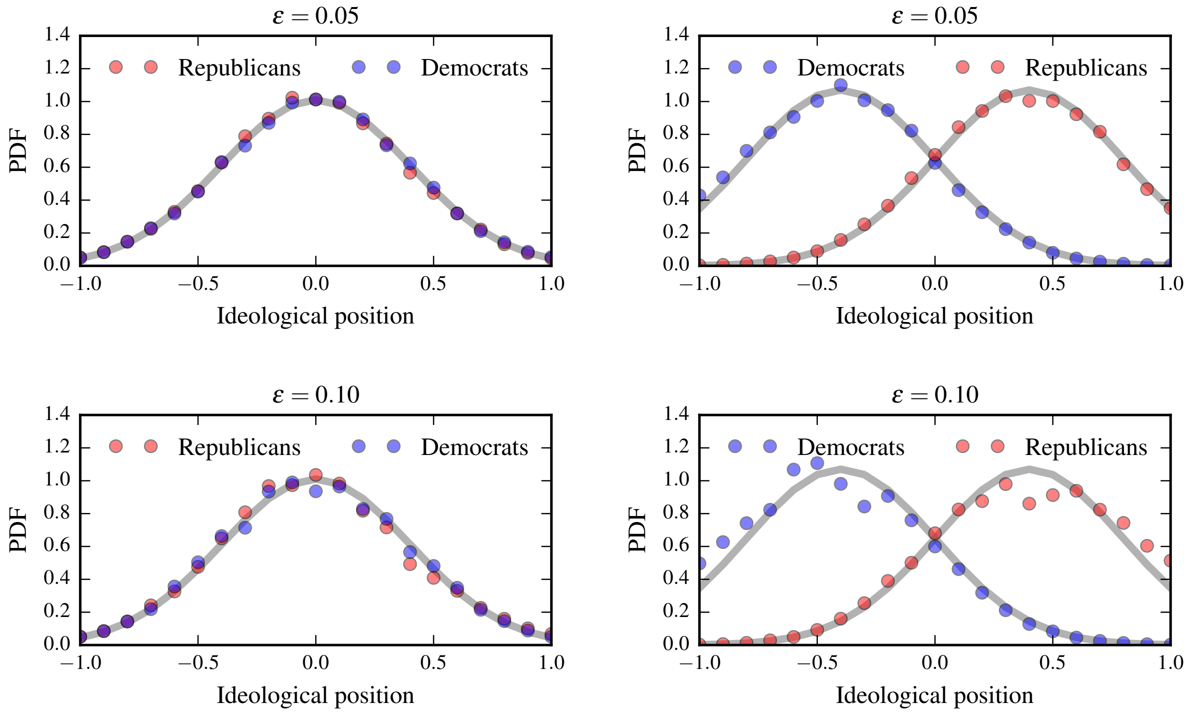

Figure S4. **Influence of noise.** We show the influence of noise effects by considering relative fluctuations in the transition probabilities p_i^A, q_i^A, p_i^B, and q_i^B ($i \in \{1, 2, \ldots, 21\}$) by an amount of ϵ. We map each value of p_i^A to $[1 + \epsilon(2u - 1)]\, p_i^A$ where $u \sim \mathcal{U}(0, 1)$ and apply the same mapping to the other transition probabilities. In the top panels, we set $\epsilon = 0.1$; and we set $\epsilon = 0.1$ in the bottom panels. The perturbed data points are represented by blue and red dots. The grey solid lines show unperturbed Gaussians. We rescaled the probabilities in the right panels according to Eqs. (10) and (11) of the main text by setting $\lambda = \lambda_A = \lambda_B = 1.13$.

[1] A. Pandey, A. Mubayi, and J. Medlock, Math. Biosci. **246**, 252 (2013).

[2] A. Gelman, H. S. Stern, J. B. Carlin, D. B. Dunson, A. Vehtari, and D. B. Rubin, *Bayesian data analysis* (Chapman and Hall/CRC, Boca Raton, FL, 2013).
